# Jejunal Leiomyoma Arising in a Diverticulum Mimicking a Hollow Viscus Perforation: A Diagnostic Challenge

**DOI:** 10.7759/cureus.115953

**Published:** 2026-09-08

**Authors:** Ajay P, Shrinidhi Katti, TP Elamurugan, Sushil Nepali

**Affiliations:** 1 General Surgery, Jawaharlal Institute of Postgraduate Medical Education and Research, Puducherry, IND; 2 Surgery, Jawaharlal Institute of Postgraduate Medical Education and Research, Puducherry, IND

**Keywords:** emergency laparotomy, gist, hollow viscus perforation, jejunal leiomyoma, small bowel tumor

## Abstract

Small intestinal leiomyomas are rare benign smooth muscle tumors that are now infrequently encountered following the reclassification of many mesenchymal tumors as gastrointestinal stromal tumors (GISTs). They are usually asymptomatic or present with nonspecific symptoms such as abdominal pain or gastrointestinal bleeding. Acute presentation with perforation peritonitis is exceedingly rare and poses a diagnostic challenge. We report the case of a 50-year-old female who presented with fever, vomiting, and acute abdominal pain with features of localized peritonitis. Contrast-enhanced computed tomography demonstrated pneumoperitoneum with a heterogeneously enhancing large outpouching arising from a mid-jejunal loop with wall thickening and internal air foci, suggestive of complicated jejunal diverticulitis or perforated neoplasm. Emergency exploratory laparotomy revealed a diverticulum-like outpouching arising from the jejunum with localized purulent contamination. Segmental jejunal resection with primary anastomosis was performed. Microscopic examination revealed a well-circumscribed spindle cell tumor arising from the muscularis propria, composed of spindle cells arranged in fascicles with mild to moderate pleomorphism, elongated to oval nuclei, vesicular chromatin, variably prominent nucleoli, and moderate cytoplasm. Focal bizarre nuclei and increased vascular proliferation were noted without necrosis or mitotic activity. Immunohistochemistry showed positivity for smooth muscle actin (SMA) and h-caldesmon, while CD117 and DOG1 were negative, confirming leiomyoma and excluding GIST. The postoperative course was uneventful. This case highlights the diagnostic difficulty associated with rare small bowel tumors presenting as acute abdomen and emphasizes the importance of surgical exploration, histopathology, and immunohistochemistry in establishing the diagnosis and guiding management.

## Introduction

Small intestinal leiomyomas are rare benign smooth muscle tumors that are now infrequently encountered, as many lesions previously classified as leiomyomas have been redefined as gastrointestinal stromal tumors (GISTs) with the advent of immunohistochemistry [1,2]. They are typically slow-growing and remain clinically silent due to their intramural or extraluminal growth pattern. Symptomatic cases usually present with nonspecific features such as abdominal pain or gastrointestinal bleeding, while acute presentations are uncommon [3,4]. Perforation peritonitis as an initial manifestation is exceptionally rare and can mimic more common surgical emergencies, posing a diagnostic challenge [5]. We report a rare case of jejunal leiomyoma presenting as perforation peritonitis, emphasizing the importance of considering unusual etiologies in patients with acute abdomen.

## Case presentation

A 50-year-old female with no known comorbidities presented to the emergency department with complaints of abdominal pain for one day, vomiting (two episodes) for one day, and fever with chills for two days. She was apparently well three days prior, when she developed a high-grade fever with chills and rigors, which subsided with medication. She also reported multiple episodes of vomiting containing food particles and not bile-stained. Additionally, she gave a history of intermittent postprandial abdominal pain for the past 15 days, which gradually progressed in severity. The patient had been diagnosed with typhoid fever one month prior and had received intravenous antibiotics at an outside hospital.

On presentation, the patient was conscious, alert, and oriented but appeared distressed due to abdominal pain. Vital signs were as follows: temperature 38.2°C, pulse rate 110 beats per minute, blood pressure 124/78 mmHg, respiratory rate 20 breaths per minute, and oxygen saturation 98% on room air. The general physical examination was unremarkable except for signs of dehydration. Abdominal examination revealed a mildly distended abdomen with a centrally placed umbilicus and no visible scars, sinuses, or dilated veins. There was localized tenderness in the right iliac fossa with rebound tenderness, without guarding or rigidity. No palpable mass or organomegaly was noted. Percussion was predominantly tympanic with no shifting dullness. Bowel sounds were sluggish on auscultation. Per rectal examination revealed hard stools were present with normal staining and no evidence of melena or fresh blood.

Our initial differential diagnoses were acute appendicitis with localized peritonitis, small bowel pathology such as diverticulitis or a contained perforation, and, in view of the recent history of typhoid fever, ileal perforation was kept in mind. The progressive postprandial abdominal pain also prompted consideration of mesenteric ischemia, though the clinical findings were not classical. Further imaging was planned to establish the diagnosis.

A chest radiograph revealed free air under the diaphragm. Laboratory investigations showed mild leukocytosis with normal renal function parameters. Ultrasonography of the abdomen revealed dilated bowel loops with sluggish peristalsis with minimal inter-bowel free fluid. Contrast-enhanced computed tomography (CECT) of the thorax and abdomen revealed a heterogeneously enhancing large outpouching arising from a mid-jejunal loop with wall thickening and internal air foci, measuring 3.9 × 3.2 × 3.8 cm. Surrounding mesenteric fat stranding and multiple perilesional air foci were also noted. Contrast opacification of the mesenteric vessels was preserved. The findings were suggestive of complicated jejunal diverticulitis with perforation, with a perforated neoplasm as a differential diagnosis (Figure 1).

**Figure 1:**
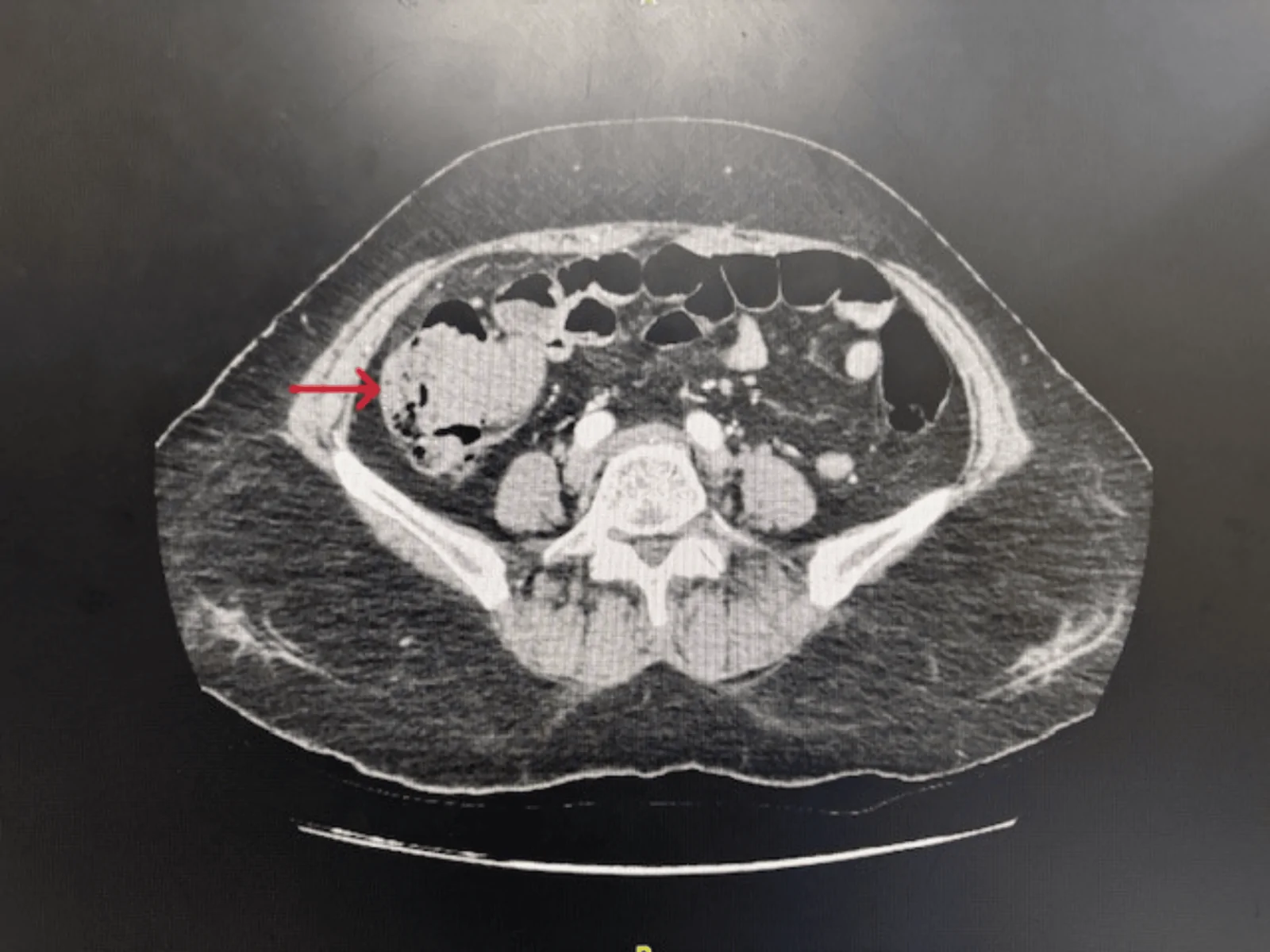
Contrast-enhanced axial CT abdomen showing localized small bowel perforation Contrast-enhanced axial CT image of the abdomen (axial section) demonstrating a heterogeneously enhancing small bowel wall lesion with perluminal air foci (red arrow) suggestive of localized perforation with associated inflammatory changes.

Free intraperitoneal air was seen anterior to the liver, consistent with pneumoperitoneum. No ascites or significant mesenteric or retroperitoneal lymphadenopathy was identified (Figure 2).

**Figure 2:**
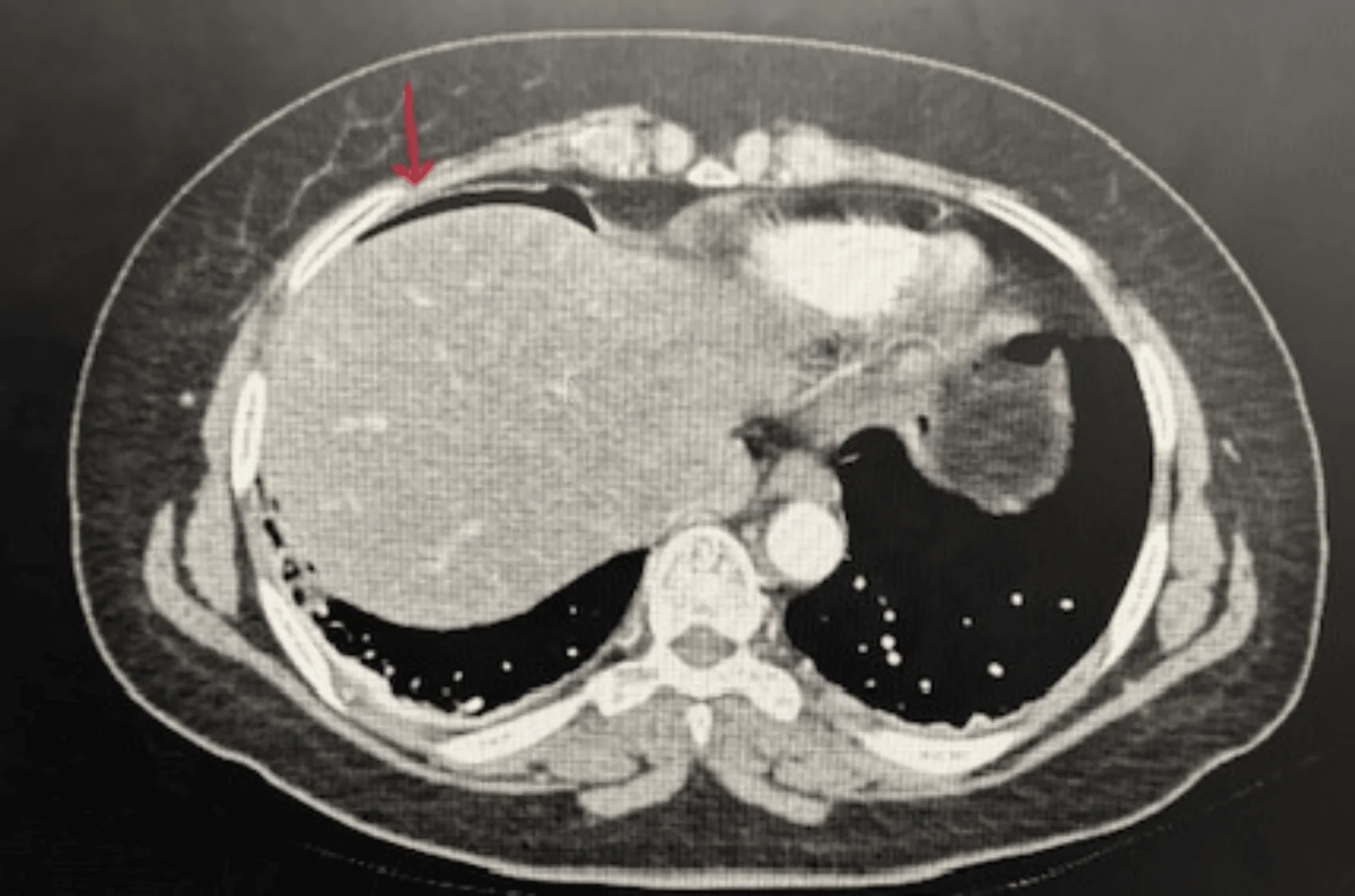
CECT abdomen showing free intraperitoneal air Contrast-enhanced computed tomography (CECT) of the abdomen (axial section) depicting free intraperitoneal air anterior to the liver (red arrow).

In view of perforation peritonitis, the patient underwent emergency exploratory laparotomy under general anesthesia. Intraoperatively, minimal purulent contamination was noted in the right lumbar region, and the collected pus was sent for culture. A diverticulum-like outpouching was identified in the jejunum approximately 140 cm distal to the duodenojejunal flexure, with surrounding pus flakes. The remaining bowel appeared normal on thorough exploration (Figure 3).

**Figure 3:**
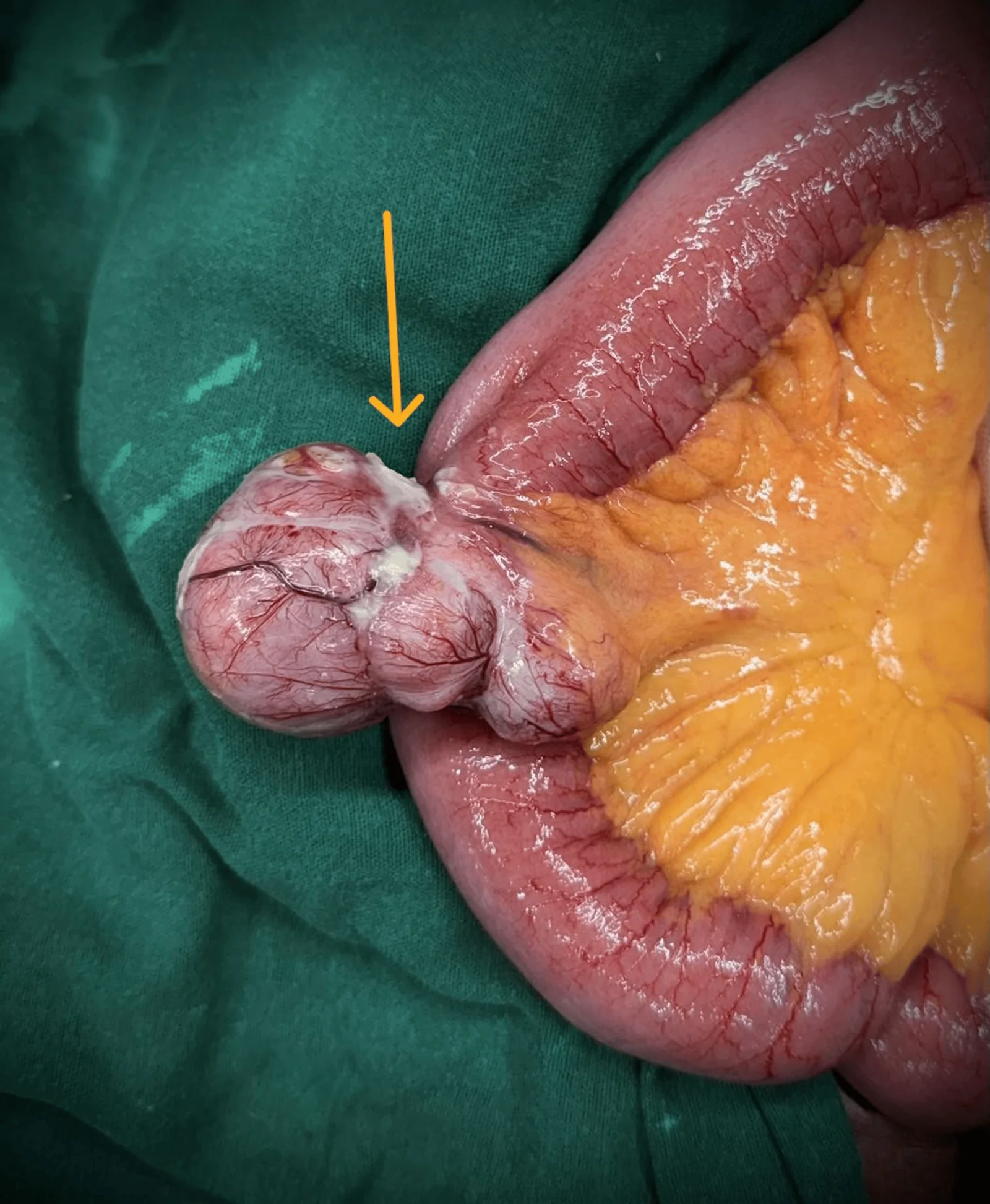
Intraoperative image demonstrating a jejunal diverticulum-like outpouching Intraoperative image showing a diverticulum-like outpouching arising from the jejunum, with a smooth, rounded serosal bulge and prominent surface vascularity.

A segmental resection of the affected jejunal segment was performed with a 10 cm margin both proximally and distally. This was followed by a hand-sewn, two-layer end-to-end anastomosis using 3-0 Vicryl and 3-0 silk sutures. Thorough peritoneal lavage was carried out with 3 liters of warm saline. A 32 Fr drain was placed in the pelvis and exteriorized through the right flank. The abdomen was closed in layers, with rectus sheath closure using 1-0 PDS in a modified Smead-Jones technique, and the skin was approximated with staples.

The patient was monitored in the recovery room for 48 hours before being shifted to the surgical ward. The immediate postoperative period was uneventful. The nasogastric tube was removed on postoperative day (POD) 2, and oral sips were initiated on POD 3, which were well tolerated and gradually advanced to a full oral diet. Drain output decreased progressively, and the drain was removed on POD 5. The patient received empirical intravenous ceftriaxone and metronidazole for five days. Intraoperative pus culture, blood culture, and sputum culture were sterile. At the time of discharge, the patient was ambulating well, tolerating oral intake, passing stools, and had a healthy surgical wound with staples in situ. At follow-up 10 days after discharge, the staples were removed, and the surgical wound was healthy with no evidence of complications.

On macroscopic examination, the specimen comprised a 20-cm segment of jejunum with attached mesentery measuring 19 × 3.5 × 1 cm. The serosal surface revealed a bosselated mass arising from the bowel wall, measuring 5.5 × 3.5 × 4 cm (Figure 4).

**Figure 4:**
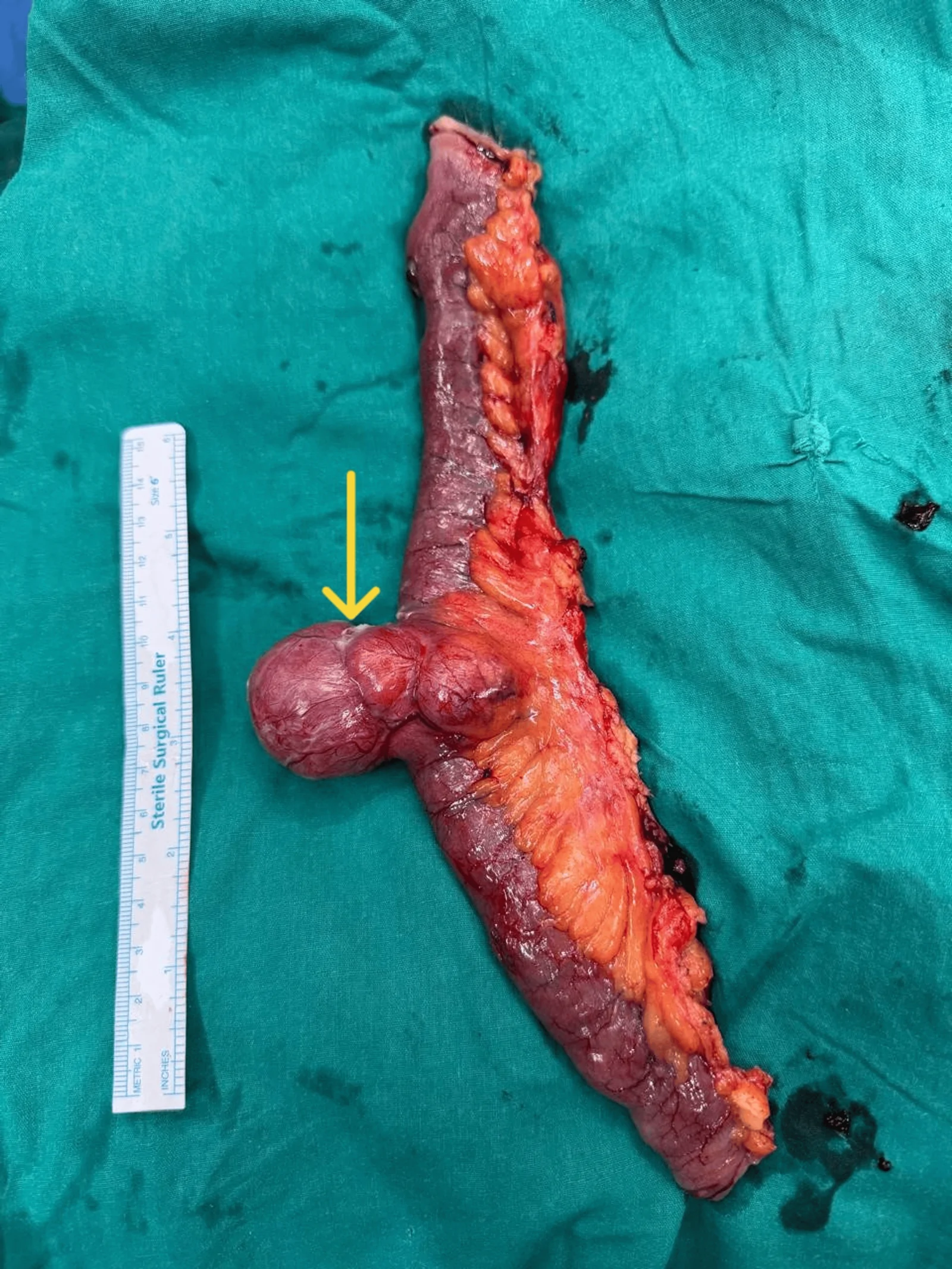
Gross specimen showing a well-circumscribed bosselated mass arising from the jejunal wall Resected jejunal segment with attached mesentery demonstrating a well-circumscribed, bosselated mass arising from the bowel wall.

On the cut section, the lesion was well-circumscribed, solid, and firm, with focal cystic degeneration involving less than 5% of the tumor. No areas of hemorrhage or necrosis were identified. The adjacent mucosa appeared unremarkable, and no definite perforation or diverticulum was identified in the resected specimen (Figure 5).

**Figure 5:**
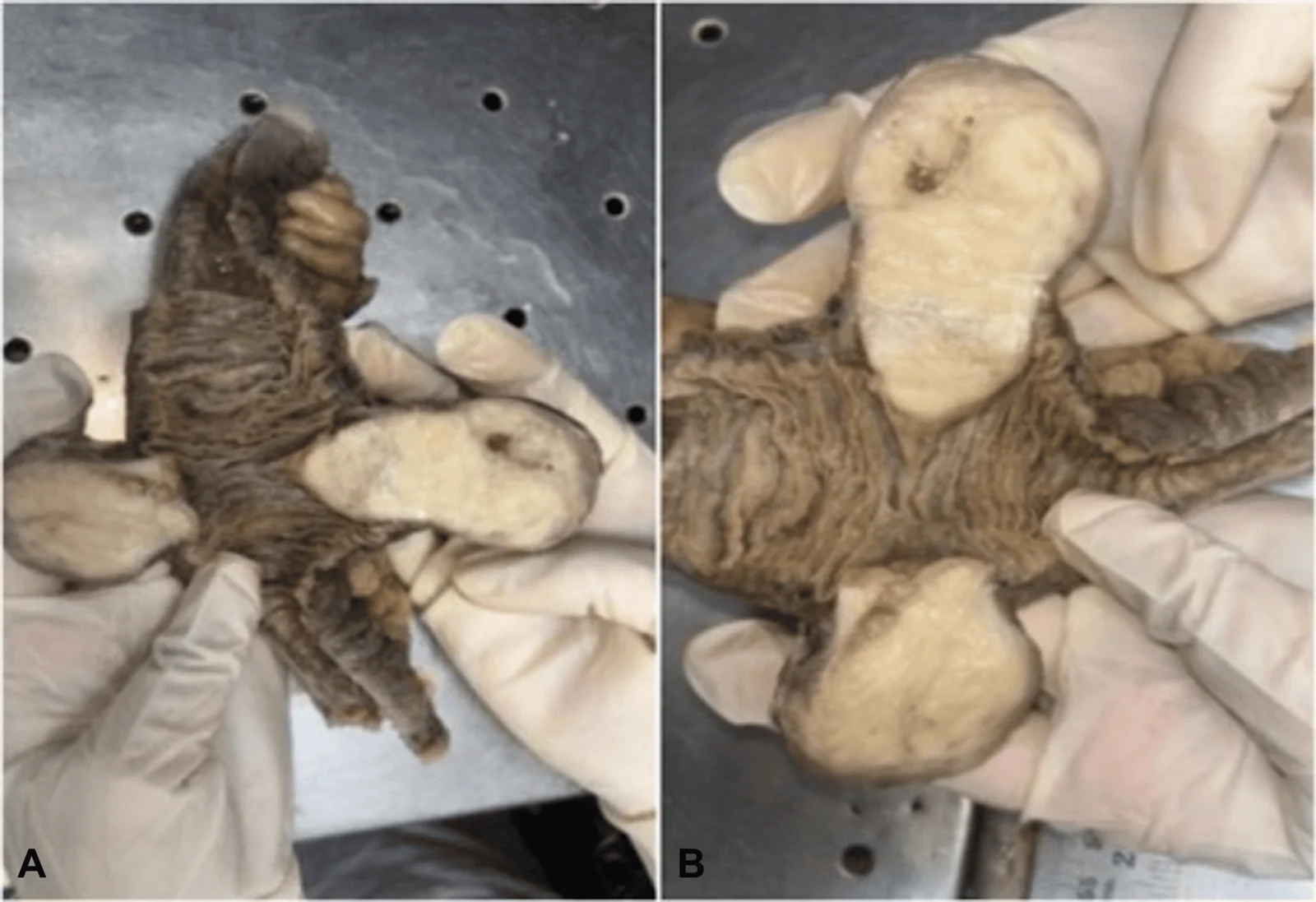
Gross pathological specimen of the resected jejunal segment (A) External surface showing a well-defined, bosselated mass arising from the bowel wall. (B) Cut section demonstrating a well-circumscribed, solid, firm lesion with a homogeneous whitish appearance and focal areas of cystic change, without evidence of hemorrhage or necrosis.

Microscopic examination revealed normal jejunal mucosa with a well-circumscribed tumor located in the muscularis propria. The tumor was composed of spindle cells arranged in fascicles, showing mild to moderate pleomorphism, elongated to oval nuclei with vesicular chromatin, variably prominent nucleoli, and moderate cytoplasm. Focal areas showed bizarre nuclei and increased vascular proliferation. No necrosis or mitotic activity was noted. The tumor cells were positive for smooth muscle actin (SMA) and h-caldesmon and negative for CD117 and DOG1.

## Discussion

Most leiomyomas follow an indolent course and remain asymptomatic for long periods; when symptoms occur, they are usually related to mucosal ulceration or mass effect, presenting as gastrointestinal bleeding, anemia, or vague abdominal pain [3,4]. Acute abdomen is an unusual presentation, with only a limited number of cases reported in the literature [5]. The present case is notable for this atypical and severe presentation. Preoperative diagnosis remains challenging due to nonspecific clinical features and the limited accessibility of the jejunum to routine endoscopic evaluation. In acute settings, CECT plays a pivotal role; however, imaging findings are often non-specific. Leiomyomas may mimic other conditions such as jejunal diverticulitis, intra-abdominal abscess, inflammatory masses, or perforated malignancy, as seen in this case. This diagnostic ambiguity frequently necessitates surgical exploration for definitive diagnosis.

The mechanism of perforation in leiomyomas is not well established. Proposed mechanisms include progressive tumor enlargement causing pressure-related ischemia of the overlying bowel wall, secondary infection, or mucosal ulceration [6]. In the present case, histopathology did not demonstrate a mucosal breach, ischemic changes, ulceration, or necrosis. Therefore, the precise mechanism underlying the perforation cannot be established. A sealed or microscopic perforation remains a possible explanation, but this is hypothetical and cannot be confirmed histopathologically. Nevertheless, the presence of significant peritoneal contamination despite the absence of an overt mucosal defect highlights the potential for perforation to occur without an identifiable mucosal breach.

Histopathological examination remains the gold standard for diagnosis. Leiomyomas are characterized by well-circumscribed spindle cell tumors arranged in interlacing fascicles, with minimal atypia, low mitotic activity, and absence of necrosis [7]. Immunohistochemistry is essential to differentiate leiomyomas from GISTs, which carry malignant potential and require different management strategies. Leiomyomas typically express smooth muscle markers such as SMA and desmin, while lacking expression of CD117 and DOG1. This distinction is critical for differentiating it from GIST, prognostication, and therapeutic planning [8].

Surgical resection remains the definitive treatment for symptomatic or complicated leiomyomas. In the setting of perforation peritonitis, emergency exploratory laparotomy with segmental resection and primary anastomosis is both diagnostic and therapeutic. Lymphadenectomy is not indicated due to the benign nature of the tumor and the negligible risk of nodal spread. Complete excision is associated with excellent outcomes and minimal risk of recurrence [3,4].

This case underscores the importance of considering rare small bowel tumors in the differential diagnosis of acute abdomen. It also highlights the limitations of preoperative diagnosis and the pivotal role of surgical intervention in both diagnosis and management. Reporting such rare presentations contributes to the existing literature and enhances clinical awareness, which is essential for timely decision-making and optimal patient outcomes.

## Conclusions

Jejunal leiomyoma is a rare benign tumor that typically follows an indolent course, with presentation as perforation peritonitis being exceptionally uncommon. This case highlights the diagnostic challenges posed by its nonspecific clinical and radiological features and emphasizes the importance of maintaining a high index of suspicion for uncommon small bowel neoplasms in patients presenting with acute abdomen. Histopathological examination with immunohistochemistry remains essential for definitive diagnosis and differentiation from GISTs. Prompt surgical resection is both diagnostic and curative, resulting in excellent outcomes. Reporting such unusual presentations expands the existing literature and aids in improving clinical recognition and management of these rare entities.
